# The molecular mechanism of microRNA duplex selectivity of *Arabidopsis* ARGONAUTE10

**DOI:** 10.1093/nar/gkac571

**Published:** 2022-07-08

**Authors:** Yao Xiao, Ian J MacRae

**Affiliations:** Department of Integrative Structural and Computational Biology, The Scripps Research Institute, La Jolla, CA 92037, USA; Department of Integrative Structural and Computational Biology, The Scripps Research Institute, La Jolla, CA 92037, USA

## Abstract

Small RNAs (sRNAs), including microRNAs (miRNAs) and small interfering RNAs (siRNAs), are essential gene regulators for plant and animal development. The loading of sRNA duplexes into the proper ARGONAUTE (AGO) protein is a key step to forming a functional silencing complex. In *Arabidopsis thaliana*, the specific loading of miR166/165 into AGO10 (AtAGO10) is critical for the maintenance of the shoot apical meristem, the source of all shoot organs, but the mechanism by which AtAGO10 distinguishes miR166/165 from other cellular miRNAs is not known. Here, we show purified AtAGO10 alone lacks loading selectivity towards miR166/165 duplexes. However, phosphate and HSP chaperone systems reshape the selectivity of AtAGO10 to its physiological substrates. A loop in the AtAGO10 central cleft is essential for recognizing specific mismatches opposite the guide strand 3′ region in miR166/165 duplexes. Replacing this loop with the equivalent loop from Homo sapiens AGO2 (HsAGO2) changes AtAGO10 miRNA loading behavior such that 3′ region mismatches are ignored and mismatches opposite the guide 5′ end instead drive loading, as in HsAGO2. Thus, this study uncovers the molecular mechanism underlying the miR166/165 selectivity of AtAGO10, essential for plant development, and provides new insights into how miRNA duplex structures are recognized for sRNA sorting.

## INTRODUCTION

Small RNAs play essential roles in regulating a wide range of physiological processes, such as embryogenesis (1), pathogen defense (2), flower patterning (3) in plants, cancer progression (4), cardiac function (5) and neuron development (6) in animals. In general, sRNA biogenesis involves cleavage of miRNA hairpin precursors (pre-miRNAs) or long double-stranded RNAs (dsRNAs) by members of the DICER protein family into 20–24-nucleotide (nt) sRNA duplexes with 5′ phosphate and 2 nt overhangs at 3′ ends on both strands (7). sRNA duplexes are then loaded into AGO proteins – the core protein component of the RNA-induced silencing complex (RISC). AGO proteins are composed of N-terminal (N), Linker-1 (L1), PAZ, Linker-2 (L2), MID and PIWI domains to form a bilobed structure with a cleft in the center to retain the sRNA (8). During the loading process, the sRNA duplex is inserted into the central cleft of the AGO protein. The 5′-phosphate of one strand (termed the guide or miRNA strand) is anchored into a conserved 5′ nucleotide-binding pocket in the MID domain. The other strand (termed the passenger or miRNA* strand) remains unanchored and is either cleaved by AGO endoribonuclease activity or unwound from the guide strand, ejected from the complex, and degraded. The anchored strand retained in the central cleft of the AGO protein is then used as a guide for finding complementary sites in transcripts targeted for repression (9). Thus, the loading process is also called RISC assembly. RISC assembly involves conformational changes in AGO proteins, and the Hsp90/Hsp70 protein chaperone systems have been found to be involved in multiple RISC assemblies to assist their conformational changes, such as *Drosophila melanogaster* AGO2 (DmAGO2) (10–12), mammalian AGOs (12–14) and AtAGO1 (15). The production rate of sRNA duplexes can exceed the rate of RISC assembly, such that RISC assembly serves as a bottleneck to control sRNA homeostasis inside cells (16,17).

RISC assembly often involves sorting specific sRNA duplexes into specific AGO proteins. Sorting can direct sRNAs to different AGO silencing mechanisms, which can function at the post-transcriptional level by direct target RNA cleavage (18), translational inhibition (19,20), and target degradation (21), or at the transcriptional level by guiding DNA and histone modification (22), or by priming the generation of secondary siRNAs (23). AGO selection can also impact sRNA stability (24). Thus, the loading step is critical for determining the fate and function of sRNAs.

Multiple factors are known to influence sRNA sorting. In human AGOs, a nucleotide specificity loop in the MID domain makes hydrogen bonds with 5′ uridine (U) or adenosine (A) but not with cytidine (C) or guanosine (G), and thereby confers a preference for guides with 5′ U and A (25). Plant AGOs also have 5′ nucleotide preferences, wherein AtAGO1 prefers sRNAs with 5′ U, AtAGO2 prefers 5′ A, and AtAGO5 favors 5′ C (26). sRNA loading also follows the thermodynamic asymmetry rule, where the strand with the less stably paired 5′ end is preferentially selected as guide (27,28). For DmAGO2, guide strand selection is influenced by the orientation of the sRNA bound to the DICER2–R2D2 complex, which recognizes sRNA duplex thermodynamic asymmetry (29). However, a requirement for the DICER2-R2D2 complex is not universal, since asymmetric loading of sRNA duplexes into mammalian AGO proteins can be recapitulated without DICER proteins and data suggest that an unstable 5′ end could favor its docking into the 5′ nucleotide-binding pocket in AGO proteins (30,31). Additionally, the internal structures of sRNA duplexes can play a role in the sorting of sRNAs into different AGOs. For example, mismatches in the middle of sRNA duplexes are responsible for the sorting between AtAGO1 and AtAGO2 in *Arabidopsis* (32) as well as for sorting between DmAGO1 and DmAGO2 in *Drosophila* (33).

*Arabidopsis thaliana* expresses two AGO proteins, AtAGO7 and AtAGO10, that display remarkably exclusive sRNA loading preferences, with each largely dedicated to one or two specific sRNAs (23,34). Approximately 90% of AtAGO10-bound miRNAs are miR166 and miR165, which differ by only one nucleotide at the 17^th^ position (counting from the 5′ end), and have identical duplex structures (34). AtAGO10 plays an essential role in the development of shoot apical meristem (SAM), which is the source of all plant shoot organs. AtAGO10 mutant plants display various defects in SAM development (35). Defects in loading miR166 into AtAGO10 cause a phenotype similar to AtAGO10 knockout (34), indicating that specific sorting of miR166 into AtAGO10 is important to its function in plant development. Curiously, AtAGO10 mutant plants have elevated levels of miR166 and miR165 as well as decreased levels of miR-165/166 target transcripts (36). Additionally, when bound to AtAGO10, miRNAs are more susceptible to the degradation by SMALL RNA DEGRADING NUCLEASES (SDNs) than when bound to AtAGO1 – the primary repressor of miRNA targets in *Arabidopsis* (24). Thus, a major, albeit noncanonical function of AtAGO10 is to bind miR165/166 and promote its degradation, thereby limiting the loading of miR165/166 into AtAGO1 and elevating the expression levels of miR165/166 targeted genes that are important to maintaining the stemness of the SAM (24,34).

Zhu *et al.* demonstrated that the preferential loading of miR166 into AtAGO10 *in vivo* is determined by the unique structure of the miR166/miR166* duplex (34). Compared to other plant miRNA/miRNA* duplexes, which typically have only 0 or 1 mismatches in the guide strand 3′ region, base pairing to the miR166 3′ region in the duplex is less stable, with mismatches at the 12th, 13th and 16th nucleotide positions. These 3′ region mispairings are important for miR166 sorting into AtAGO10, as a miR166 duplex variant that was mutated to be stable in the miR166 3′ region had compromised loading into AtAGO10. Moreover, another miR166 duplex variant, having stable pairing at the miR166 5′ end, was loaded into AtAGO10 with wild-type efficiency (34). Thus, miR166 loading seemingly defies the thermodynamic asymmetry rule, wherein sRNA duplex stability at the 5′ end of the guide strand usually reduces its loading efficiency. The mechanism underlying stringent recognition of instability in 3′ half of the miR166 duplex by AtAGO10, as well as the factors involved, are unknown. Therefore, to better understand this unique and important aspect of plant miRNA biology, as well as gain broader insight into sRNA sorting mechanisms, we developed a reconstituted biochemical system to dissect the miRNA selectivity behavior of AtAGO10.

## MATERIALS AND METHODS

### Oligonucleotides

#### miR166 and its passenger strands

miR166: 5′- Phosphate-rUrCrGrGrArCrCrArGrGrCrUrUrCrArUrUrCrCrCrC-3′

miR166-WT-passenger: 5′- Phosphate-rGrGrArCrUrGrUrUrGrUrCrUrGrGrCrUrCrGrArGrG-3′

miR166-mutant-1-passenger: 5′- Phosphate-rGrGrArCrUrGrUrUrGrUrCrUrGrGrUrCrCrGrArGrG-3′

miR166-mutant-2-passenger: 5′- Phosphate-rGrGrArArUrGrArArUrCrCrUrGrGrCrUrCrGrArGrG-3′

#### Template and promoter for in vitro transcription

miR166_T19 DNA template:

5′-mGmGCCTCGGACCAGGCTTCATTCCGCCTATAGTGAGTCGTATTA-3′

T7 promoter sequence: 5′-TAATACGACTCACTATAGGC-3′

#### In vitro transcription of miR166_T19

For annealing template: mix 10μl 100μM T7 promoter and 10μl 100μM miR166_T19 DNA template with 5μl 5x annealing buffer (50 mM Tris (pH 8), 250mM NaCl). Heat the sample at 95°C for 10 min, and let it naturally cool down to RT on the bench. 1:5 dilute the annealed template as working concentration. Transcription reaction: 30 μl diluted template, 60 μl NTPs mix (25 mM ATP + UTP + CTP + GTP), 60 μl 10× Transcription Buffer (400 mM Tris 8, 60 mM MgCl_2_, 100 mM DTT, 20 mM Spermidine), 54 μl 100 mM MgCl_2_, 15 μl Murine RNase Inhibitor, 15 μl T7 RNA Pol, add H_2_O up to 600 μl. Incubate at 37°C for 3 h, then add 30 μl DNase I and incubate for another 1 h. Add 600 μl phenol/chloroform (low pH), vortex it, centrifuge at 15 000 × g for 5 min, and transfer the aqueous phase to a new EP tube. Add 1 μl glycogen, 1/10 vol. 3 M NaOAc, and 3× vol. 100% EtOH, precipitate RNA at −20°C for ∼1 h. Pellet RNA at 15 000 × g in the cold room for 10min, wash the pellet with 1 ml 75% EtOH and add 60 μl EB to re-suspend the pellet. Then, gel purify it by running a 15% Urea PAGE gel and using UV shadow to identify the band. The band is cut out and crushed, then, soak in ∼500 ul crush and soak buffer @cold room rocker overnight. The next day, the gel is filtered by a Spin-X tube. Add 1 μl glycogen, 1/10 vol. 3 M NaOAc and 3× vol 100% EtOH into the filtered solution, precipitate RNA at −20°C for ∼1 h. Pellet RNA at 15 000 × g in the cold room for 10min, wash the pellet with 75% EtOH, add ∼50 μl EB to re-suspend the pellet, and quantify the RNA by nanodrop.

### G-cap radiolabel miR166_T19

miR166_T19 are G-cap radiolabeled by the vaccinia capping system (NEB, Cat.# M2080S). Briefly, ∼1.5 μg gel-purified *in vitro* transcribed T19 RNA is incubated with 4 μl α-^32^P-GTP, 3 μl 10× capping buffer, 1.5 μl 2 mM SAM, 1.5 μl capping Enzyme, and 17 μl H_2_O at 37°C for 30 min. The radiolabeled target was then gel purified.

### Construction of AtAGO10 constructs

Full-length Arabidopsis AtAGO10 (AtAGO10) cDNA was amplified from an AtAGO10 encoded plasmid (a gift from Dr Yingnan Hou and Dr Wenrong He), and cloned into a pFastBac-His-Flag-TEV (pFB-HFT) vector. The plasmids were transformed into DH10Bac chemically competent *Escherichia coli* (ThermoFisher) for Bacmid DNA preparation. The mutations in the second central loop were introduced by amplifying two separate AtAGO10 coding sequence fragments with overlap at the mutation region using the primers including mutated sequence, and then, these two fragments were assembled together with the pFB-HFT vector using NEBuilder® HiFi DNA Assembly Master Mix (Cat. #E2621S).

### Expression and purification of AtAGO10

His6-Flag-Tev-tagged AtAGO10 protein was expressed in Sf9 cells using a baculovirus expression system (Invitrogen). Four 750ml of 3.4 × 10^6^ cells/ml Sf9 cells were infected with 13–15 ml amplified virus at 27°C for ∼60 h and then harvested by centrifugation. Cell pellets were resuspended in Lysis Buffer (50 mM NaH_2_PO_4_ (pH 8), 300 mM NaCl, 5% glycerol, 20 mM imidazole, 1 mM PMSF, 0.5 mM TCEP) and lysed by an M-110P lab homogenizer (Microfluidizer, Westwood, MA). The resulting total cell lysate was clarified by centrifugation (30 000 × g for 25 min) and the soluble fraction was applied to 8 ml packed Ni-NTA resin (Qiagen) and incubated at 4°C for 1.5 h in 50 ml conical tubes. Ni-NTA resin was pelleted by brief centrifugation and the supernatant solution was discarded. The resin was washed 3 times with ∼50 ml ice cold Nickel Wash Buffer (50 mM Tris(pH 8), 300 mM NaCl, 20 mM imidazole, 0.5 mM TCEP). Bulk cellular RNAs were then degraded by on-resin treatment with 800 units of microccocal nuclease (Clontech). The nuclease-treated resin was washed three times again with Nickel Wash Buffer and then eluted by applying two sequential two-column volumes of Nickel Elution Buffer (50 mM Tris (pH 8), 300 mM NaCl, 300 mM imidazole, 0.5 mM TCEP). ∼150 ug TEV protease was incubated with the eluted sample during a ∼3 h dialysis against 1 l dialyzing buffer (50 mM Tris(pH 8), 300 mM NaCl, 0.5 mM TCEP), then moved to a fresh 2 l dialyzing buffer to dialyze overnight. On the second day, precipitation in dialyzed protein was pelleted by centrifugation at 3000 × g for 5 min. The supernatant was applied to a 5 ml HisTrap column (GE Life Sciences) equilibrated with dialyzing buffer, and the fraction containing AtAGO10 protein was eluted by dialyzing buffer with 30 mM Imidazole. This fraction was then further purified by size exclusion chromatography using Superdex 200 Increase 10/300 GL column (GE Life Sciences). The peak fractions containing AtAGO10 were combined and dialyzed against 1 l of Tris Crystal Buffer (10 mM Tris (pH 8), 100 mM NaCl, 0.5 mM TCEP) for 4 h to overnight. Lastly, the dialyzed protein was concentrated to 5–6 mg/ml in Tris Crystal Buffer and stored at –80°C.

### Construction of Chaperone constructs

The CDS of AtHsp90.3 (AT5G56010.1), AtHsp70 (AT3G12580.1), AtHop (AT1G62740.1), AtDnaJ2 (AT5G22060.1) and AtP23 (AT4G02450.1) were amplified from Col-0 cDNA (a gift from Dr Wenrong He and Dr Ye Xu), and cloned into pET23a-His-TEV vector.

### Expression and purification of Chaperone proteins

All pET23a-His-TEV plasmids encoding Chaperone proteins were transformed into Rosetta™(DE3) Competent Cells (Novagen) for expression. Briefly, 1 l bacteria cultures were grown at 37°C to an *A*_600_ of 0.8, and protein production was induced with 0.4mM Isopropyl β-d-1-thiogalactopyranoside (IPTG), followed by growth overnight at 16°C. Cells were harvested by centrifugation, and cell pellets were resuspended in lysis buffer (50 mM NaH_2_PO_4_ (pH 8), 300mM NaCl, 5% glycerol, 30 mM imidazole, 1 mM PMSF, 0.5 mM TCEP) and lysed by an M-110P lab homogenizer (Microfluidics, Westwood, MA). The resulting total cell lysate was clarified by centrifugation (30 000 × g for 25 min) and the soluble fraction was applied to 1.5 ml packed Ni-NTA resin (Qiagen) and incubated at 4°C for 1.5 h in 50 mL conical tubes. The resin was pelleted by brief centrifugation and the supernatant solution was discarded. The resin was washed 3 times with ∼50 ml ice-cold Nickel Wash Buffer (50 mM Tris(pH 8),300 mM NaCl, 30 mM imidazole, 0.5 mM TCEP). Bulk cellular RNAs were then degraded by on-resin treatment with 100 units of micrococcal nuclease (Clontech). The nuclease-treated resin was washed three times again with Nickel Wash Buffer and then eluted in four column volumes of Nickel Elution Buffer (50 mM Tris (pH 8), 300 mM NaCl, 300 mM imidazole, 0.5 mM TCEP). ∼150 ug TEV protease was incubated with the eluted sample during overnight dialysis against 1 l dialyzing buffer (50 mM Tris(pH 8), 300 mM NaCl, 30mM imidazole, 0.5 mM TCEP). The protein was then passed over a second Ni-NTA column, and the unbound fraction was collected and further purified using size exclusion chromatography. Purified Chaperone proteins (except for AtDnaJ2) were concentrated in 10 mM Tris (pH 8.0), 0.1 M NaCl, and 0.5 mM TCEP to 5–10 mg/ml and stored at −80°C. AtDnaJ2 was concentrated in 50 mM Tris (pH 8.0), 0.3 M NaCl and 0.5 mM TCEP.

### RISC assembly and target slicing assay in Sf9 lysate

Sf9 cells with either AtAGO10 or HsAGO2 expressed were resuspended in 2× volume of lysis buffer (30 mM HEPES pH(7.5), 100 mM KOAc, 5 mM MgOAc, 1 mM DTT, 1× cOmplete™, EDTA-free Protease Inhibitor Cocktail Tablets), and homogenized by ∼30 strokes with Dounce homogenizer. The insoluble cell debris was pelleted at max speed by sequential 5 and 10 min spins using a benchtop centrifuge in the cold room, the supernatant was kept for assays. Then, 7.5 μl supernatant was incubated with 0.5 μl 20× ATP-regeneration system (20 mM ATP, 0.6 U/ul Creatine Kinase, 2 U/ul RNasin Plus), 0.2 μl 1M Creatine Phosphate, 1.8 μl lysis buffer and 5 μl 100 nM Guide RNA at RT for 1h. After incubation, 1 μl 40nM G-cap* miR166_T19 was added to the reaction and incubated at RT for 10 min. The reaction was quenched by 16 μl 2× FLB. The samples were examined by 15% urea PAGE, the gel was exposed to a phosphor screen overnight, and the screen was imaged using a Typhoon scanner.

### Stability test of RNA duplex for XRN-1 treatment

Duplexes were made by mixing equal molar miR166 and passenger strands in 10 mM Tris (pH 8), 50mM NaCl, and annealed with a PCR program (95°C 2 min, 80°C 10 s, 75°C 10 s, 70°C 10 s, 65°C 30 s, 60°C 30 s, 55°C 10 s, 50°C 10 s, 45°C 10 s, 40°C 10 s, 35°C 10 s, 30°C 10 s, 25°C 10 s, 20°C 10 s, 15°C 10 s, 12°C forever). 100 nM miR166ss or duplexes were incubated with 105 nM Xrn-1 in 1× reaction buffer (30 mM HEPES (pH 7.5), 100 mM KOAc, 5 mM MgOAc, 1 mM DTT) at RT for 20 min. The samples were examined by 15% native PAGE, and stained by SYBR Gold (Invitrogen, Cat.# S11494).

### RISC assembly and target slicing assay in a purified system

1× Reaction buffer: 30 mM HEPES (pH 7.5), 100 mM KOAc, 5 mM MgOAc, 1 mM DTT. 100 nM RNA duplex was pre-incubated with 105 nM Xrn-1 in 1× Reaction buffer at RT for 20 min, miR166ss was pre-incubated with 1× Reaction buffer at RT for 20 min. For testing different small chemical compounds, 10 μl pre-treated RNA sample, 10 μl purified AtAGO10 sample with different small chemical (0.5 μM AtAGO10, 4.65 μM GST, 20 mM Chemical compound, and 0.2 U/ul RNasin Plus (Promega) in 1× Reaction Buffer) was incubated for 1 h at RT for RISC assembly. After the assembly, 1 μl of 40nM G-cap radiolabeled miR166-T19 was added to the reaction to further incubate at RT for 30 min. Finally, the reaction was quenched by 21 μl 2× FLB with 1 μl 0.5 g/l tRNA, and examined by 15% urea PAGE. For the reaction including chaperones with no ATP condition, 10 μl pre-treated RNA sample, 10 μl purified protein sample (0.5 μM AtAGO10, 0.6 μM Hsp90, 0.6 μM Hop, 0.6 μM p23, 1.2 μM Hsp70, 3 μM DnaJ2 and 0.2 U/ul RNasin Plus (Promega) in 1× Reaction buffer) was incubated for 1 h at RT for RISC assembly. For GST control, chaperone protein in the reaction was replaced by GST. For conditions with ATP-regeneration system, 10ul pretreated RNA sample, 10ul purified protein-ATP sample (0.5 μM AtAGO10 or HsAGO2, 0.6 μM Hsp90, 0.6 μM Hop, 0.6 μM p23, 1.2 μM Hsp70, 3 μM DnaJ2, 2 mM ATP, 50 mM creatine phosphate, 0.06 U/ul creatine kinase and 0.2 U/ul RNasin Plus (Promega) in 1× Reaction buffer) was incubated @RT for 1 h. After the assembly, 1 μl of 40 nM G-cap radiolabeled miR166-T19 was added into the reaction to further incubate at RT for 30min. Finally, the reaction was quenched by 21 μl 2× FLB with 1 μl 0.5 g/l tRNA, and examined by 15% urea PAGE.

### Stability test of RNA duplex for Chaperone incubation

100 nM RNA duplexes were pre-incubated with 105 nM Xrn-1 at RT for 20 min. Then, 10 μl pre-treated RNA sample was incubated with 10 μl four different types of protein samples: GST-only(4.65 μM GST, 0.2 U/ul RNasin Plus), GST + ATP (4.65 μM GST, 2 mM ATP, 50 mM creatine monophosphate, 0.06 U/ul creatine kinase, 0.2 U/ul RNasin Plus), Chaperones-only (0.6 μM Hsp90, 0.6 μM Hop, 0.6 μM p23, 1.2 μM Hsp70, 3 μM DnaJ2, 0.2 U/ul RNasin Plus) and Chaperones + ATP (0.6 μM Hsp90, 0.6 μM Hop, 0.6 μM p23, 1.2 μM Hsp70, 3 μM DnaJ2, 2 mM ATP, 50 mM creatine monophosphate, 0.06 U/ul creatine kinase, 0.2 U/ul RNasin Plus) at RT for 1 h. The reactions were quenched by 2× FLB. The samples were examined by 15% denaturing gel, and stained by SYBR-Gold (Invitrogen, Cat.# S11494).

### Filter-binding assay

miR166 was 5′-^32^P radiolabeled using a PNK exchange reaction (Thermo Scientific, cat.# EK0031). Reaction for duplexes annealing: 10 μl 10 μM radiolabeled miR166, 10 μl 10 μM cold passenger RNA, 5 μl 5× annealing buffer (50 mM Tris (pH 8), 250 °mM NaCl). The reaction was using a PCR program (95°C 2 min, 80°C 10 s, 75°C 10 s, 70°C 10 s, 65°C 30 s, 60°C 30 s, 55°C 10 s, 50°C 10 s, 45°C 10 s, 40°C 10 s, 35°C 10 s, 30°C 10 s, 25°C 10 s, 20°C 10 s, 15°C 10 s, 12°C forever). Briefly, 100 nM radiolabeled RNA duplexes was pre-incubated with 105 nM Xrn-1 at RT for 20 min, 50 μl pre-treated RNA sample was incubated with 50 μl 2-fold dilution protein samples (the highest conc: AtAGO10 is 0.5 μM, Hsp90 system is 0.6 μM Hsp90, 0.6 μM Hop and 0.6 μM p23, Hsp70 system is 1.2 μM Hsp70 and 3 μM DnaJ2) in binding reaction buffer (30 mM HEPES pH 7.5, 100 mM KOAc, 5 mM MgOAc, 0.5 mM TCEP, 0.005% NP-40) at RT for 1 h. After incubation, the samples were applied to a dot-blot apparatus with two layers of membrane, where the first layer Protran nitrocellulose membrane (0.45 μm pore size, Whatman, GE Healthcare Life Sciences) could capture protein as well as protein-RNA complex, the second layer Hybond Nylon membrane (GE Healthcare Life Sciences) could capture unbound RNA. The membranes were immediately washed once with 200 μl of ice-cold wash buffer (30 mM HEPES pH 7.5, 100 mM KOAc, 5 mM MgOAc, 0.5 mM TCEP). Membranes were air-dried and the ^32^P signal was visualized by phosphor-imaging.

## RESULTS

### miRNA duplex selectivity of AtAGO10 can be reconstituted in animal cell lysate

**Figure 1.**
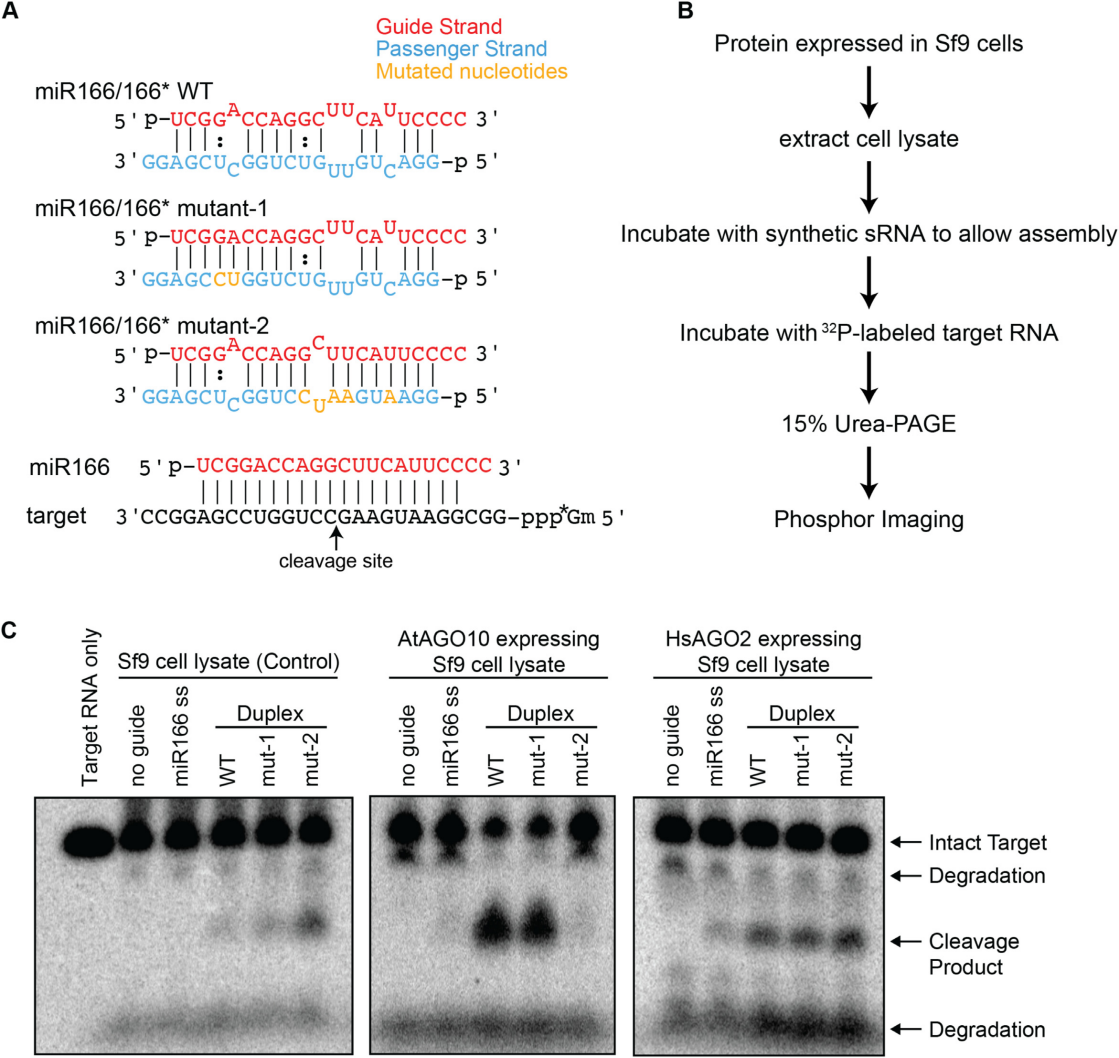
Unique miRNA duplex selectivity of AtAGO10 can be recapitulated in an animal cell lysate. (**A**) Schematics of miR166 duplex variants and miR166 paired to the miR-166 target RNA used in this study. (**B**) Flow chart of miRNA loading assay in Sf9 cell lysate. (**C**) Denaturing PAGE of ^32^P-labeled target RNAs treated with Sf9 lysates from cells expressing different AGO proteins expressed and incubated with different sRNAs. ‘miR166 ss’: single strand miR166, mut-1: miR166/166* mutant-1, mut-2: miR166/166* mutant-2.

We first asked if miRNA duplex selectivity is maintained for AtAGO10 loading in a heterologous system. AtAGO10 was overexpressed in Sf9 cells, derived from the armyworm *Spodoptera frugiperda*, using a baculovirus expression system. Infected cells were lysed, and synthetic miR166/miR166* duplexes were incubated in the soluble cell lysate. miRNA loading was assessed by observing the cleavage of an ^m7^G-capped ^32^P-labeled target RNA with complementarity to nucleotides 1–19 of miR166 (Figure 1A, B). Because animals do not possess a miR166 gene, the target RNA should be cleaved only if the exogenous miR166 is loaded into an AGO protein. To observe miRNA selectivity we also used two variants of the miR166 duplex that have differential loading properties *in vivo* (34). The mutant-1 duplex (composed of miR166 paired to miR166* C15U U16C) has increased pairing towards the 5′ end of miR166 and is loaded into AtAGO10 as efficiently as wild type (WT) miR166 duplex *in vivo*. In contrast, the mutant-2 duplex (composed of miR166 paired to miR166* C4A U7A U8A G9U U10C) has increased pairing towards the 3′ end of miR166, resembling a typical miRNA duplex, and is a very poor substrate for AtAGO10 loading in the plant (34).

Consistent with previous results in plants (34), target cleavage was observed in AtAGO10-containing Sf9 lysates incubated with WT or mutant-1 duplexes, but not mutant-2 duplex (Figure 1C, middle panel). Pulldown assays using duplexes containing ^32^P-labeled miR-166, miR-166* (wild type), or miR166* C4A U7A U8A G9U U10C (mutant-2) strands indicate that selection occurs primarily at the duplex insertion step and that passenger strand ejection is not impaired during loading of the WT and mutant-2 miR-166 duplexes (Supplementary Figure S1A). In a control lysate made from Sf9 cells overexpressing human AGO2, all three variants of the miR166 duplex induced target cleavage (Figure 1C right panel). An additional control, uninfected Sf9 cell lysate, displayed a moderate level of target cleavage when incubated with the mutant-2 duplex and low levels with WT and mutant-1 (Figure 1C, left panel), likely the results of loading into AGO proteins endogenous to Sf9 cells. Single-stranded miR166 induced only weak target cleavage in all lysates, possibly because the 5′-phosphorylated single-stranded RNA is susceptible to rapid degradation by exonuclease activity in the lysate prior to loading. A version of the miR-166 duplex based on the duplex structure of miR-168, which has more extensive complementarity and is not loaded into AtAGO10 *in vivo* (34), produced even less active AtAGO10-miR166 complex than mutant-2 (Supplementary Figure S1B). The combined results indicate the specific duplex selectivity of AtAGO10 can be recapitulated biochemically. Moreover, faithful selectivity by AtAGO10 can occur in a completely heterologous system.

### Purified AtAGO10 displays a different duplex preference

**Figure 2.**
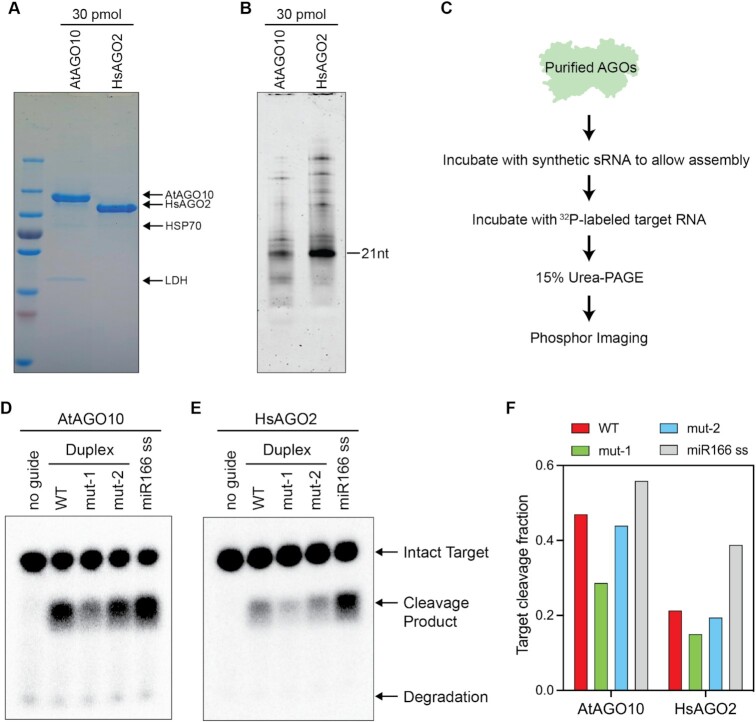
Purification of AtAGO10 removes *in vivo* miRNA duplex preferences. (**A**) The purity of recombinant AtAGO10 and HsAGO2 proteins was analyzed by SDS-PAGE followed by Coomassie blue staining. (**B**) Equal molar amounts of AtAGO10 and HsAGO2 preparations were analyzed by 15% urea–PAGE stained with SYBR-Gold to show the relative sizes and amounts of co-purifying nucleic acids. (**C**) Flow chart of miRNA loading assay using purified protein. Target cleavage by (**D**) purified AtAGO10 and (**E**) purified HsAGO2 loaded with different miRNA species. (**F**) Quantification of the fraction of target RNA cleaved in (D) and (E).

Observing faithful duplex selectivity of AtAGO10 in animal cell lysate indicated that either: (a) AtAGO10 is the sole factor required for selectivity; or, (b) any additional selectivity factors are present in both plant and animal cells. To distinguish between these possibilities, we examined the biochemical properties of purified AtAGO10. Recombinant AtAGO10 was purified to near homogeneity from Sf9 cells with two minor protein contaminants identified as Sf9 HSP70 family member and lactate dehydrogenase (LDH) by mass spectrometry (Figure 2A). Staining for RNA present in equivalent amounts of purified AtAGO10 and HsAGO2 protein demonstrated less RNA co-purified with AtAgo10, indicating that more AtAGO10 was purified in an apo (unloaded) state than HsAGO2 (Figure 2B).

A semi-quantitative AtAGO10 and miRNA assembly assay was established to assess the loading efficiency of different miR166 duplexes (Figure 2C). To minimize confounding effects from residual single strand guide RNA, annealed duplexes were pre-incubated with purified XRN1, which efficiently degrades single-stranded miR166, but acts slowly on miR166 duplexes (Supplementary Figure S2A). XRN1-treated miR166 duplexes (or an untreated single-stranded miR166 control) were incubated with purified AtAGO10 at room temperature for 1 h to allow AtAGO10-miR166 assembly. The radiolabeled target was then added to the reaction and incubated for an additional 30 min to allow the target cleavage to reach a plateau (Supplementary Figure S2B). Serial dilutions of the assembly reaction demonstrated that the observed fraction of cleaved target molecules was proportional to the amount of assembled AtAGO10-miR166 present in the reaction mixture (Supplementary Figure S2C).

Incubation of purified AtAGO10 with either WT or mutant-2 duplex led to similar levels of target cleavage. By contrast, the mutant-1 duplex assembly reaction produced a lower amount of active AtAGO10-miR166 complex (Figure 2D, F). This result reveals that purified AtAGO10 favors mutant-2 duplex over mutant-1, which is the opposite of that observed in Sf9 lysate (Figure 1C), and previously reported in plants (34). In fact, purified HsAGO2 displayed a similar duplex selection pattern (Figure 2E, F), revealing that, once purified, both AtAGO10 and HsAGO2 prefer miRNA duplexes with unstable structure near the guide 5′ end. This result indicated that additional factors, present in both Sf9 cell lysate and in plant cells, reshape duplex selection by AtAGO10.

### Phosphate and sulfate inhibit AtAGO10 loading and modulate miRNA duplex selectivity

The discrepancy of miRNA duplex preference between AtAGO10 in lysate and purified AtAGO10 suggested additional factor(s), present in both plant and animal cells, reshape the duplex selectivity of AtAGO10. The Hsp70/Hsp90 chaperone machinery was a likely candidate because these proteins are conserved among eukaryotes and function in RISC-assembly in both plants (15) and animals (10–14). Biochemical reconstitution of chaperone activity typically includes an ATP-regeneration system (such as 1 mM ATP, 25 mM creatine phosphate (CP), and Creatine Kinase (CK)) (Supplementary Figure S3A) (10,37). We, therefore, first carried out control reactions containing the ATP-regeneration system (without any chaperone proteins). To our surprise, the ATP-regeneration system alone partially reversed duplex selectivity by AtAGO10, with more target cleavage in the mutant-1 assembly reaction than mutant-2 (Supplementary Figure S3B,C). The change in duplex selectivity was accompanied by a reduction in cleaved products in all samples. These effects were variable between experiments and largely diminished when using freshly prepared ATP and Creatine phosphate (Supplementary Figure S3B,C), signifying a breakdown product, such as free phosphate produced by hydrolysis of ATP or CP, could be the cause of the observed changes in loading efficiency.

**Figure 3.**
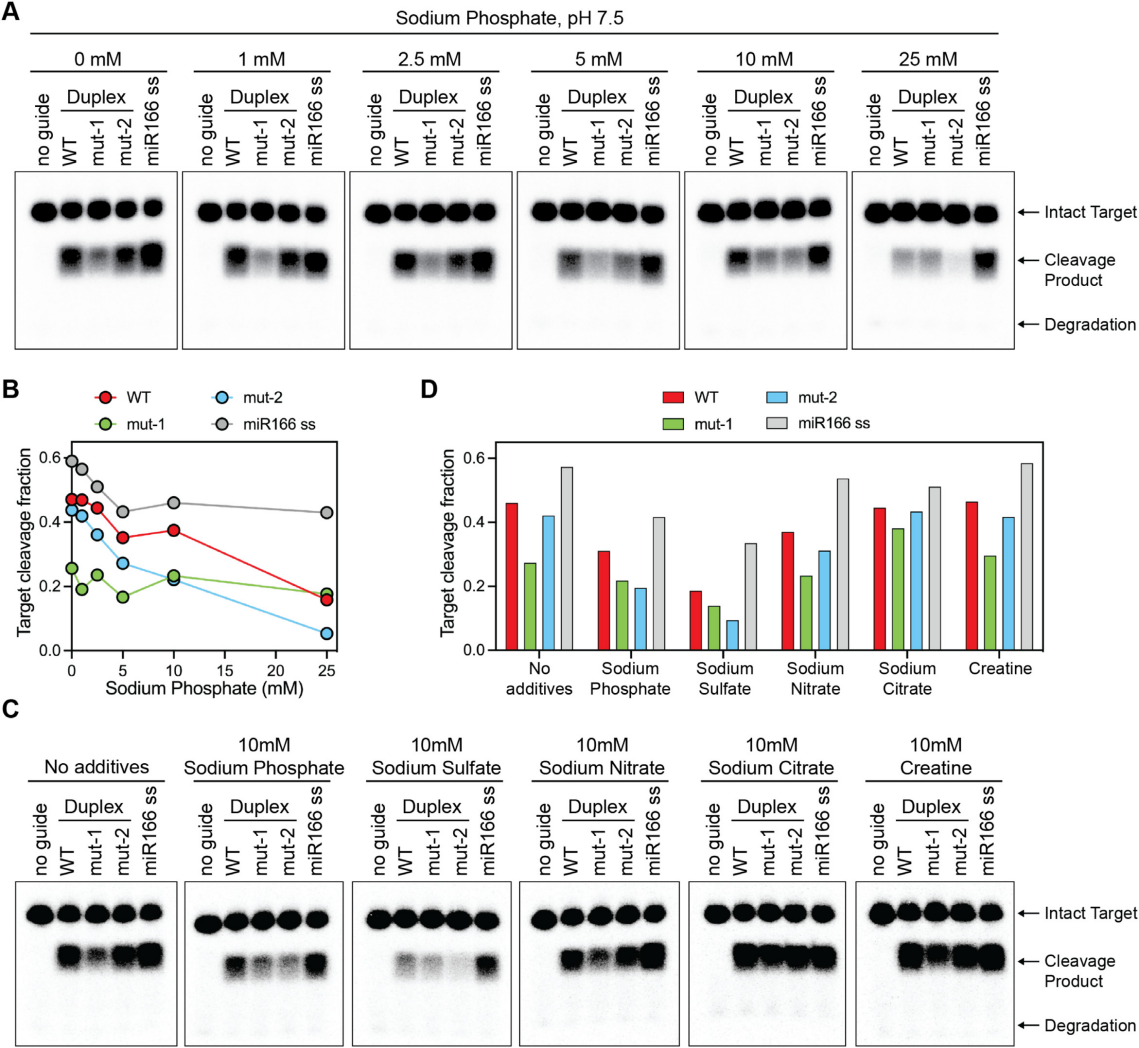
Phosphate and sulfate modulate miRNA duplex selectivity by disproportionally inhibiting mut-2 duplex loading. (**A**) Target cleavage by AtAGO10-miR166 assembled with various concentrations of sodium phosphate. (**B**) Quantification of fraction target RNA cleaved in (A). (**C**) Target cleavage by AtAGO10-miR166 assembled with various additives. (**D**) Quantification of target cleavage in (C).

To explore this idea, we examined the effects of inorganic phosphate using our AtAGO10-miR166 assembly assay. We found that inhibition increased with increasing concentrations of sodium phosphate (Figure 3A, B). Moreover, ≥10 mM phosphate shifted AtAGO10 duplex selectivity from resembling human AGO2 (WT = mutant-2 > mutant-1) towards AtAGO10 *in vivo* (WT > = mutant-1 > mutant-2) (Figure 3A). Phosphate was not inhibitory when added to the reaction mixture between the loading and slicing steps (Supplementary Figure S4), demonstrating phosphate mainly modulates the loading of AtAGO10, but not its slicing activity. We also examined the effects of 10 mM sodium chloride, sodium sulfate, sodium nitrate, creatine, l-glutamate and d-glucose. Nitrate, l-glutamate and d-glucose had almost no effect on the assay (Figure 3C, D, and Supplementary Figure S5). Sodium chloride had modest inhibitory effects but did not change duplex selectivity preferences (Supplementary Figure S5). Most notably, sodium sulfate induced duplex selectivity changes similar to those caused by sodium phosphate (Figure 3C, D, and Supplementary Figure S5).

The combined results demonstrate that AtAGO10 loading selectivity and efficiency are modulated by phosphate and sulfate, both of which are tetrahedral oxyanions. Importantly, phosphate and sulfate strongly inhibit the loading of the mutant-2 duplex and have more moderate effects on the loading of WT and mutant-1 duplexes (Figure 3A, B). Thus, by disproportionally inhibiting the loading of mutant-2, these oxyanions switch the miRNA duplex preference of AtAGO10. HsAGO2-miRNA assembly was also inhibited by phosphate but, in contrast to AtAGO10, the duplex preference of HsAGO2 is not affected (Supplementary Figure S6).

### HSP70 and HSP90 systems have distinct yet coordinated effects on miRNA duplex loading and selectivity of AtAGO10

Including phosphate shifted purified AtAGO10 towards the guide selection pattern observed *in vivo*, but also reduced overall guide-loading efficiency. We therefore moved on to analyze the effects of Hsp90 and Hsp70 chaperone systems. We cloned and purified recombinant versions of the following *Arabidopsis* chaperones: AtHSP90.3 (AT5G56010.1), AtHSP70 (AT3G12580.1) and co-Chaperones AtHOP (AT1G62740.1), AtDnaJ2 (AT5G22060.1), AtP23 (AT4G02450.1)) (Supplementary Figure S7A).

**Figure 4.**
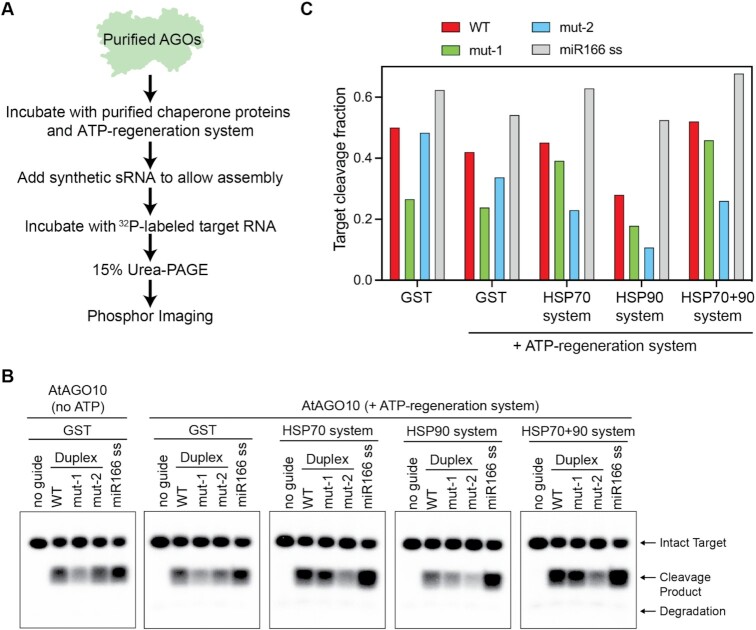
HSP chaperone systems reshape the miRNA duplex selectivity of AtAGO10. (**A**) Flow chart of the AtAGO10-miRNA assembly assay with chaperone proteins included in the system. (**B**) Target cleavage by AtAGO10-miR166 assembled with different chaperone system components. (**C**) Quantification of target cleavage fraction in (B).

In the presence of the ATP-regeneration system, the HSP70 system (HSP70 + DnaJ2) increased the loading of WT and mut-1 duplexes, while concurrently diminishing the loading of mut-2 compared to a GST control (Figure 4), resulting in a duplex selectivity pattern similar to *in vivo*. By contrast, the HSP90 system (HSP90 + HOP + P23) inhibited the loading of all three miRNA duplexes into AtAGO10 with mut-2 duplex loading more strongly inhibited than WT and mut-1 duplexes (Figure 4). Notably, inhibition of mut-2 loading by the HSP90 system was observed even without ATP, (Supplementary Figure S7), revealing the HSP90 system alone helps prevent incorrect duplex loading. Intriguingly, combining the HSP90 system with the HSP70 system further stimulated the loading of WT and mut-1 duplex compared to the HSP70 system alone, while maintaining a low loading of mut-2 duplex (Figure 4B, C). The stimulatory effect of the HSP90 system in the presence of the HSP70 system is subtle but repeatable among different batches of purified AtAGO10 and chaperone proteins. Thus, the full combination of the HSP70 system, HSP90 system, and ATP-regeneration system reshapes the duplex selectivity of AtAGO10 to a pattern very similar to as observed *in vivo*.

We also assessed interactions between the HSP systems, AtAgo10, and input miR-166 duplex RNA. Pull-down assays showed that AtAGO10 directly interacts with AtHSP70 and AtHSP90 (Supplementary Figure S8B–E). By contrast, the chaperone system proteins did not interact with or degrade RNA duplexes (Supplementary Figure S9). Thus, the chaperone systems exert their effects by influencing AtAGO10 conformation and not by affecting the RNA duplexes differently.

### A specialized central cleft loop is necessary for AtAGO10 duplex selectivity

**Figure 5.**
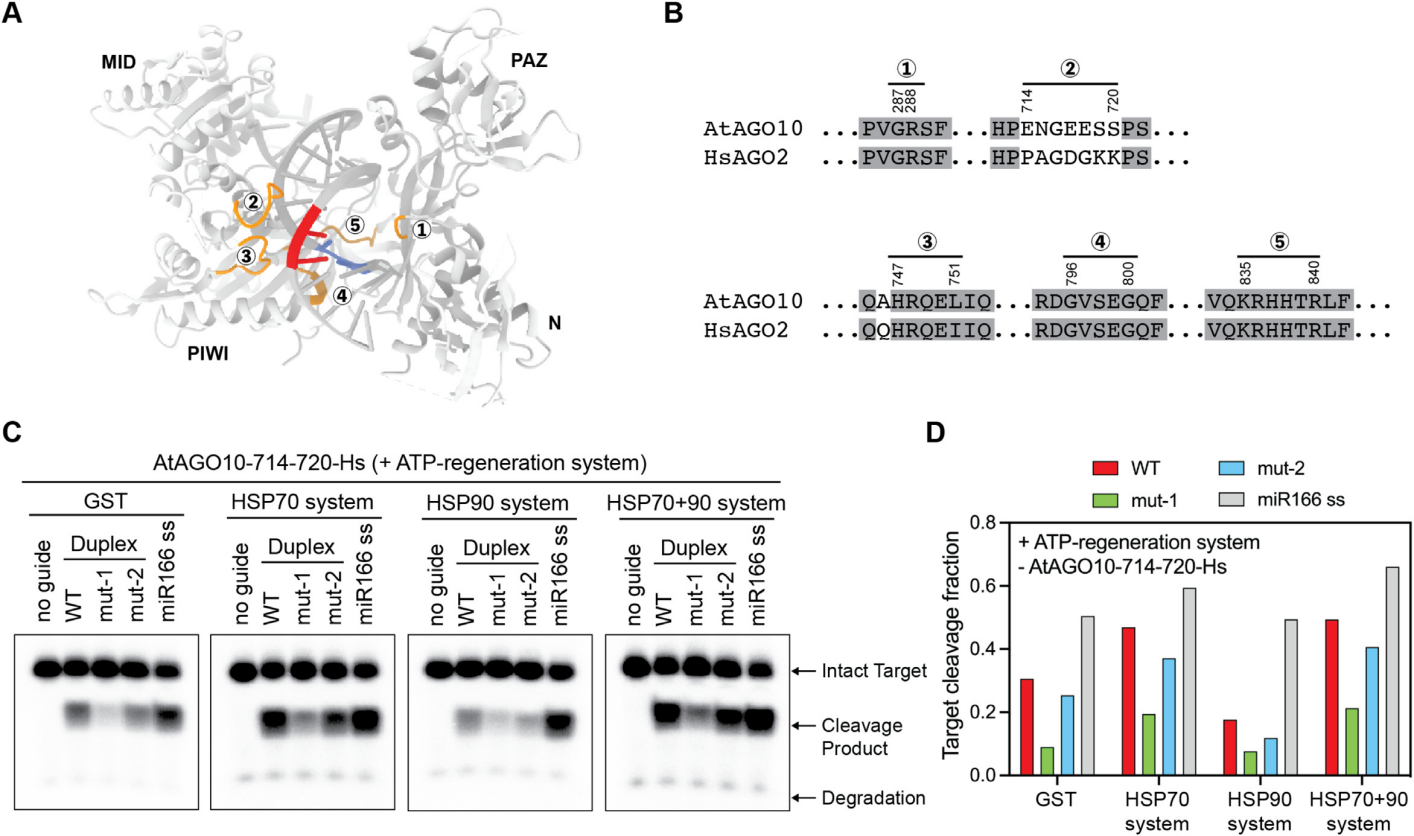
A central loop participates in the special duplex selectivity of AtAGO10. (**A**) Ribbon drawing of the AtAGO10 structure (PDB: 7SWF) with five central cleft loops highlighted (orange color) near nucleotides 12 and 13 of the guide (red) and passenger (blue) strands. (**B**) Sequence alignment of the 5 loops from AtAGO10 and HsAGO2. (**C**) Target cleavage by AtAGO10-714-720-Hs (amino acid residues 714–720 in AtAGO10 replaced with the corresponding sequence in HsAGO2) assembled with miR166 and different chaperone system components. (**D**) Quantification of target cleavage fraction in (C).

In contrast to the duplex selectivity shift observed for AtAGO10, the duplex selectivity of HsAGO2 was not changed by including chaperone and ATP-regeneration systems in the assembly reaction (Supplementary Figure S10). This observation suggests that AtAGO10 possesses specific features within its structure to recognize different miRNA duplex structures. A recently determined AtAGO10–miRNA–target complex cryo-EM structure (PDB: 7SWF), where an RNA duplex docks into the central cleft of AtAGO10 and potentially mimics an intermediate of AtAGO10-miRNA assembly, opened further possibilities for exploring this idea (Figure 5A).

Mismatches at positions 12 and 13 of the miR166 diplex are important for miR166 recognition by AtAGO10 in vivo (34). Examining the AtAGO10 structure, we identified five loops in the central cleft (first loop: residues 287–288, second loop: residues 714–720, third loop: residues 747–751, fourth loop: residues 796–800, fifth loop: residues 835–840) that are located near miRNA duplex base pairs involving guide strand nucleotides 12 and 13 (Figure 5A). Thus, we hypothesized that one or more of these loops may participate in the recognition of the 12–13 mismatches. Aligning the primary sequences of HsAGO2 and AtAGO10 revealed that the second central loop is divergent, while the other four loops are highly conserved (Figure 5B). Thus, we suspected that the second loop might be responsible for the difference in duplex selectivity between AtAGO10 and HsAGO2.

We tested this hypothesis by replacing the second central loop in AtAGO10 (residues 714–720) with the sequence of the corresponding loop in HsAGO2, and expressing and purifying the resulting AtAGO10 mutant protein (AtAGO10-714-720-Hs). Like wild-type AtAGO10, the purified AtAGO10-714-720-Hs mutant prefers the mut-2 duplex over mut-1, and loading efficiency is increased by the action of the HSP chaperone systems. However, unlike wild-type, duplex selectivity preferences of the AtAGO10-714-720-Hs mutant are not altered by the chaperones (Figure 5C, D). Instead, the duplex selectivity of the AtAGO10-714-720-Hs mutant in the presence of chaperones is similar to HsAGO2 (Supplementary Figure S10). Thus, the second central loop plays an essential role in establishing the specialized duplex selectivity of AtAGO10, possibly through direct recognition of the mismatched 12–13 region of miR166/165 duplexes.

## DISCUSSION

Purified AtAGO10 displays a duplex selectivity similar to animal AGOs, which preferentially loads sRNA duplexes with an unstable guide strand 5′ end, regardless of mismatches in the 3′ guide region (Figure 2). This duplex selectivity is the opposite of that observed for AtAGO10 previously *in vivo* (34) and in Sf9 lysate (Figure 1). This observation provided us the opportunity to identify factors that reshape AtAGO10 duplex selectivity. Through rational deduction and biochemical experiments, our data suggest that the combined effects of free phosphate/sulfate and chaperone systems restrict AtAGO10 selectivity to its biologically relevant miRNA duplex.

The phosphate and sulfate concentrations that affect duplex AtAGO10-miRNA loading and selectivity (Figure 3), are within the physiological ranges of phosphate and sulfate in plant cells (1–10 mM) (38–40). Therefore, it is possible phosphate and sulfate levels *in vivo* contribute to the regulation of miR-165/166 and miR-165/166 targets in the SAM. Free phosphate also specifies the substrate selectivity of *Drosophila* DICER2, which recognizes the binding of terminal 5′ phosphates on dsRNA substrates using a pocket in the DICER2 PAZ domain (37,41). We therefore suggest that utilizing the physiological concentration of phosphate to achieve substrate selectivity might be a common mechanism for nucleic acid binding proteins whose function involves anchoring terminal phosphate groups.

Although phosphate and sulfate alone shift *in vitro* loading preferences of AtAGO10, overall loading efficiency is also reduced (Figure 3). A similar inhibitory effect was seen for the loading of HsAGO2 (Supplementary Figure S6). Therefore, we suspect that the inhibitory effect of phosphate arises from competition for a conserved site(s) in AGOs that interacts with the RNA phosphate backbone. The 5′ nucleotide-binding pocket in the MID domain (25) and seed-binding residues of the PIWI domain (8,42) are likely candidates as these regions recognize the phosphate backbone of guide RNAs during assembly of AGO-guide complexes (30). Notably, both regions interact with the 5′ end of the guide RNA, which may be connected to why loading of the mut-2 duplex is most strongly inhibited (see below).

In flies, the HSP70 system partially stimulates DmAGO2 to adopt an open conformation (43). The HSP90 system does not alter DmAGO2 conformation or loading on its own, but it does stabilize the open conformation of DmAGO2 when the HSP70 system is present (43). Our results indicate that plant chaperone systems may influence AtAGO10 in a related manner. The HSP70 system alone promotes the loading of proper miRNA duplexes, consistent with the notion that HSP70 helps AtAGO10 adopt an open conformation necessary for loading the bulky miRNA duplex, and the HSP90 system further stimulated loading (Figure 4B, C). However, in contrast to DmAGO2 results, the HSP90 system alone inhibits AtAGO10 loading, both with and without ATP (Supplementary Figure S7). Considering HSP90 can bind AtAGO10 without ATP (Supplementary Figure S8D,E), we propose that HSP90 binds apo-AtAGO10 and either sterically blocks duplex loading or pushes AtAGO10 towards a closed conformation. In the presence of the HSP70 system, however, the HSP90 system changes function to instead promote the loading of the two miRNA duplexes with proper mismatches at the guide 3′ region (Figure 4). Consistent with this idea, immobilized AtAGO10 simultaneously pulls down both HSP90 and HSP70 (Supplementary Figure S8C). Thus, chaperone-assisted AtAGO10 loading is a variation on the DmAGO2 model, where the HSP70 system primes the open conformation of AGO protein, and HSP90 stabilizes the open conformation (43).

An additional effect of the chaperone system on AGO-duplex assembly that appears specific to AtAGO10 is the chaperone system also helps AtAGO10 to achieve its special duplex selectivity (Figure 4). Notably, this effect requires the AtAGO10 central loop (residues 714–720) (Figure 5). We therefore reason that, in addition to opening the AtAGO10 central cleft to enable duplex loading, the chaperon system also positions the central loop for recognition of the miRNA duplex near the guide 3′ region.

**Figure 6.**
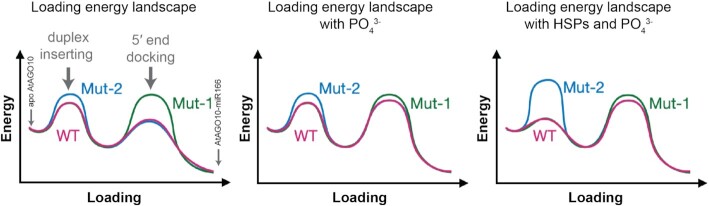
The loading energy landscape model of AtAGO10-duplex assembly under different conditions. Note: although duplex inserting is shown as the first step, the order of the two steps may be exchanged without changing the model.

Based on our results, we propose that AtAGO10 miRNA duplex-loading involves at least two discrete steps. The first step is the insertion of the miRNA duplex into the central cleft, which can be regulated by the recognition of guide 3′ region mismatches by the AtAGO10 central loop. The second step is docking the guide strand 5′ end into the AtAGO10 5′ nucleotide-binding pocket, which is enabled by fraying of the miRNA duplex and recognition of the guide 5′ phosphate (30). We suspect that, like DmAGO2 (43), purified AtAGO10 alone may adopt numerous conformations such that the central loop is often not positioned to recognize 3′ region structure. Thus, for AtAGO10 alone, the first step is energetically similar for the three duplexes (WT, mut-1, and mut-2) used in our experiments (Figure 6, left panel). Meanwhile, the less stable guide 5′ ends of WT and mut-2 duplexes enable fraying and thereby reduce the energy barrier for the second step compared to the mut-1 duplex. Thus, for AtAGO10 alone, the 5′ end docking step plays a major role in determining duplex loading efficiency.

In the case of AtAGO10 with phosphate or sulfate, the loading pattern changes to favor miRNA duplexes with 3′ region mismatches (Figure 3). In this situation, the free phosphates may compete with the guide 5′ end for the 5′ nucleotide-binding pocket, increasing the energy barrier at the second step and reducing overall loading efficiency. Inhibition is most pronounced for the mut-2 duplex, which lacks 3′ region mismatches and thus is not recognized by the central loop.

Finally, the action of chaperones shifts AtAGO10 loading to strongly favor miRNA duplexes with 3′ region mismatches. We imagine chaperones hold AtAGO10 in an open conformation, increasing loading efficiency, and position the central loop to interrogate RNA duplex structure at the guide 3′ region, increasing loading selectivity. This effectively lowers the energy barrier for inserting duplexes with proper mismatches at the guide strand 3′ region (WT and mut-1) and increases the barrier for the duplex without these mismatches (mut-2) (Figure 6, right panel). Thus, by adjusting these two energy barriers, free phosphate and the chaperone systems reshape the duplex selectivity of AtAGO10 to specifically load miR166/165 family members and ignore most other miRNAs in the plant. In future studies, it will be of interest to determine the mechanism by which the central loop discerns duplex 3′ region structure, if similar mechanisms are employed in other sRNA/AGO sorting systems, and the structural basis by which chaperones position the central loop and open the AGO central cleft.

## DATA AVAILABILITY

Plasmids carrying wild-type AtAGO10 and the mutant AtAGO10-714-720-Hs are available upon request.

## Supplementary Material

gkac571_Supplemental_File
