# Supplementary material for: The molecular mechanism of microRNA duplex selectivity of Arabidopsis ARGONAUTE10
Source: Nucleic Acids Res. 2022 Jul 8;50(17):10041–52. doi: 10.1093/nar/gkac571 (PMC9508841; doi:10.1093/nar/gkac571)
Supplement: gkac571_Supplemental_File [file gkac571_supplemental_file.docx]

**Supplementary Figures**


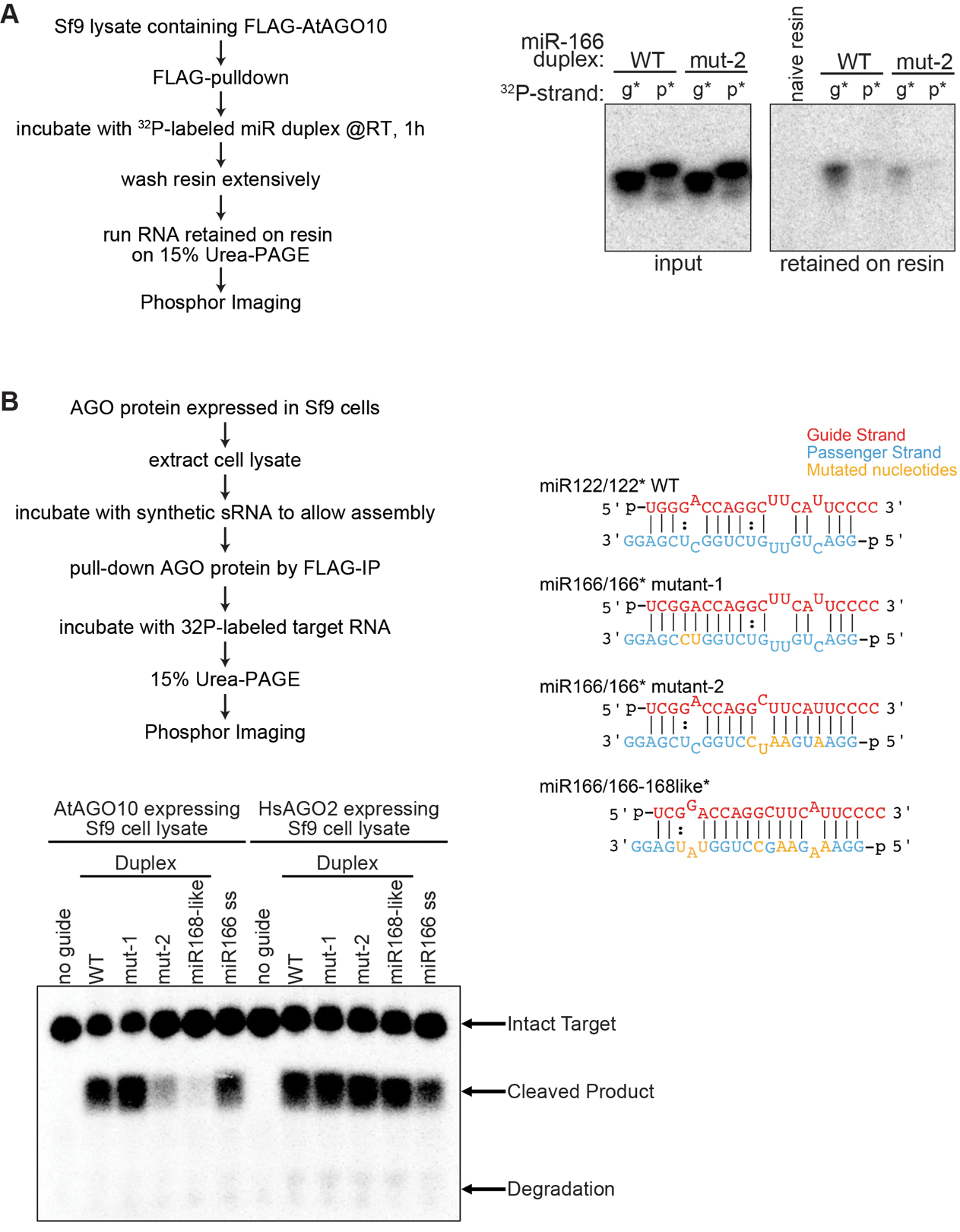


**Fig. S1. Additional assessments of duplex selection (A)** WT and mut-2 miR-166 duplexes with either guide (g) or passenger (p) strand ^32^P-labeled were incubated with FLAG resin-immobilized AtAGO10. The guide strand was preferentially retained on the resin for both duplexes after washing, indicating passenger strand ejection is not impaired and thus selection occurs primarily at the duplex insertion step. (**B**) Duplex selectivity of AtAGO10 in Sf9 lysate. Top left: flow chart of miRNA loading assay in Sf9 cell lysate. Right: schematics of miR166 duplex variants (right). Bottom: denaturing PAGE of ^32^P-labeled target RNAs treated with Sf9 lysates from cells expressing AtAGO10 or HsAGO2 incubated with sRNAs shown in the schematic. miR166/166* mutant-2 and miR166/166-168like* (as well as natural miR-168/miR-168*) are excluded from AtAGO10 *in vivo* (34).


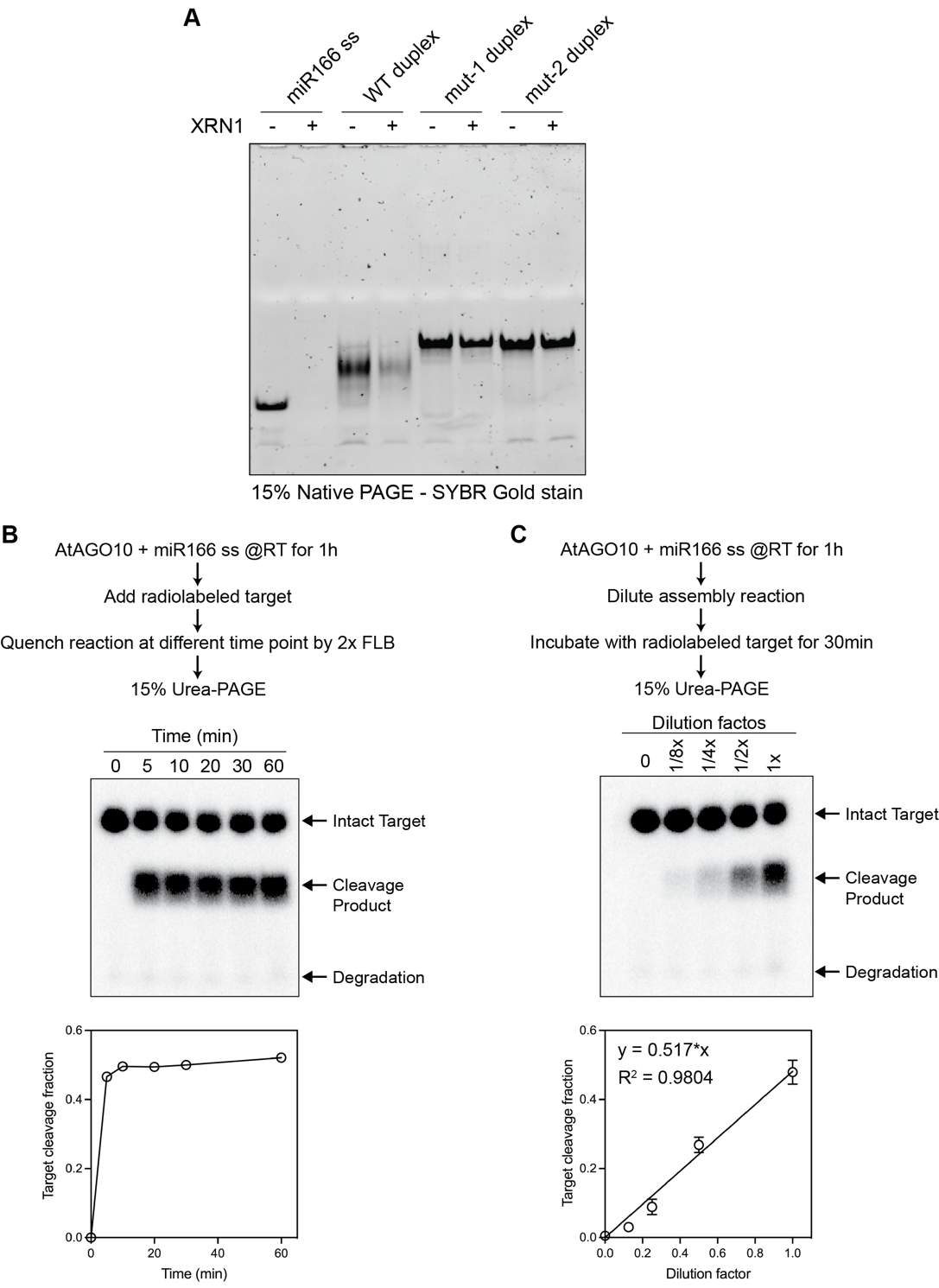


**Fig. S2. Quality control of *in vitro* duplex loading assay. (A)** RNA stability test after XRN1 treatment. ^32^P-labeled single-stranded miR166 and miR166 duplexes were incubated with XRN1 and then resolved by denaturing PAGE. **(B)** The cleavage of target RNA by assembled AtAGO10-miR166 complex over time. Results show the reaction reaches a plateau in <30 minutes. **(C)** The cleavage of target RNA by a series dilution of AtAGO10-miR166 assembly reaction. Results show the plateau value after 30 minutes is proportional to the amount of AtAGO10-guide complex added to the slicing reaction.


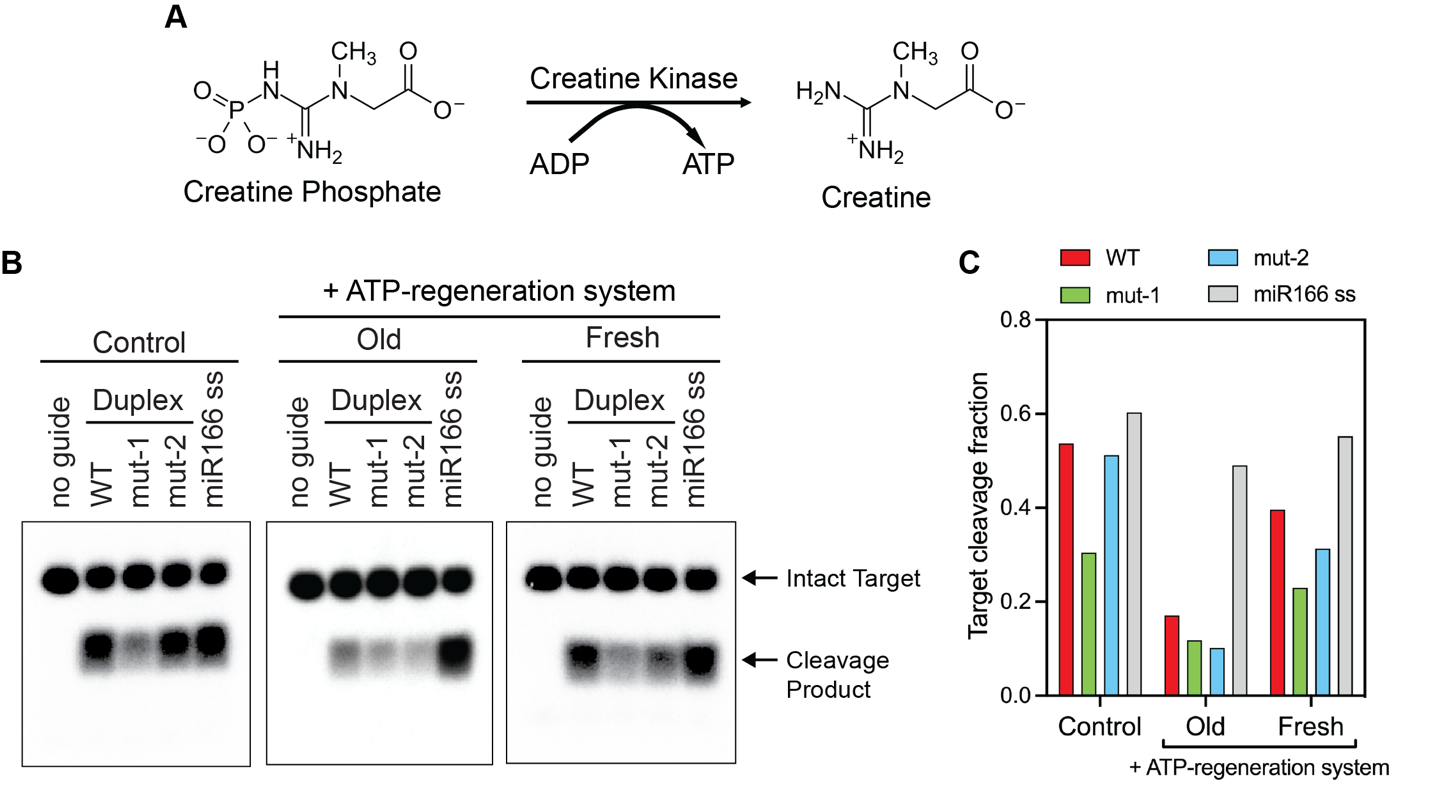


**Fig. S3. The variable result of fresh and old ATP regeneration system. (A)** Schematic of the ATP-regeneration system used in this study. **(B)** Denaturing PAGE of target RNA cleavage reactions after *in vitro* assembly of AtAGO10-miR166 in the presence of old or fresh ATP regeneration system components. **(C)** Quantification of results in panel B.


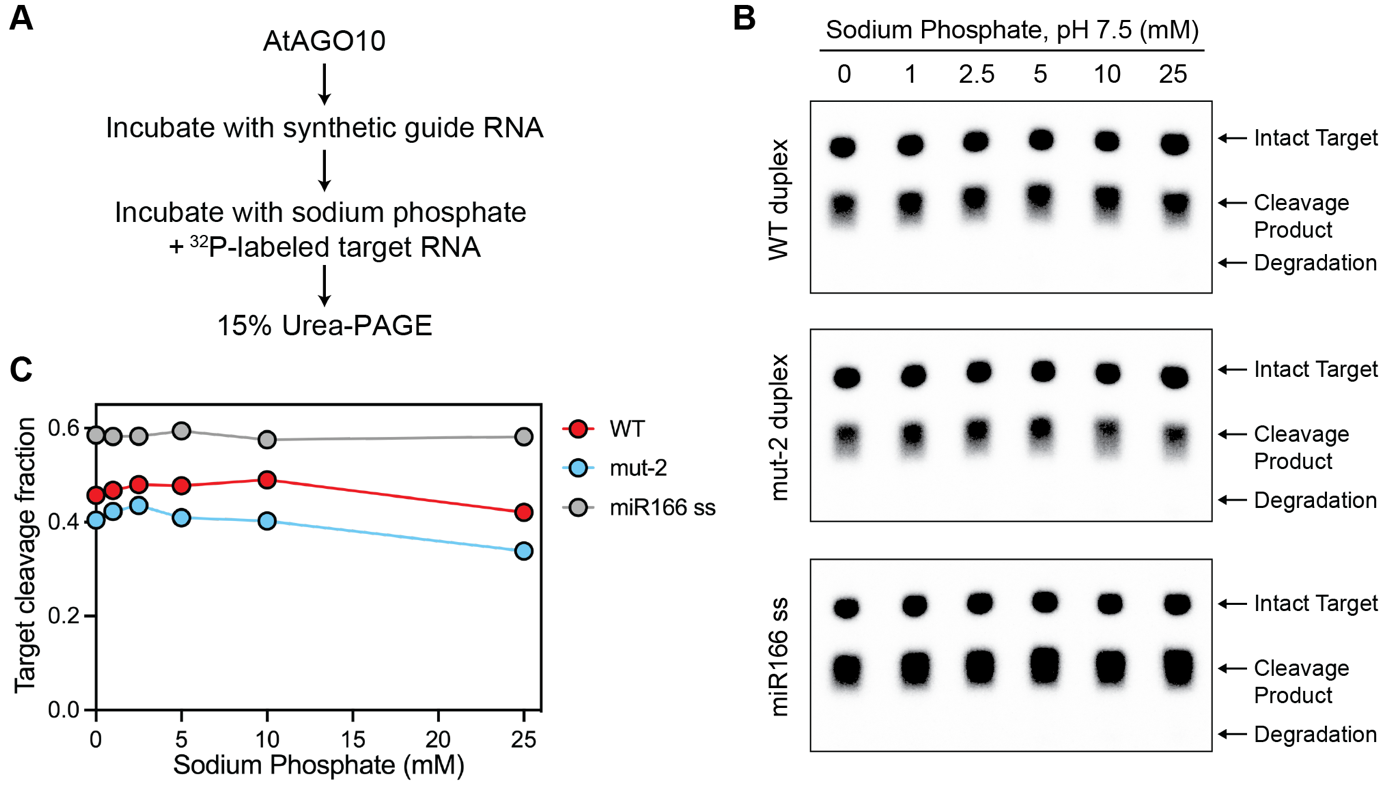


**Fig. S4. Physiological phosphate levels do not affect target slicing activity after formation of the AtAGO10-miR166 complex. (A)** Flow chart of the experimental design. **(B)** Denaturing PAGE of target cleavage reaction samples using preformed AtAGO10-miR166 complex with different concentrations of sodium phosphate (added after the assembly reaction) collected over time. **(C)** Quantification of bands in panel B.


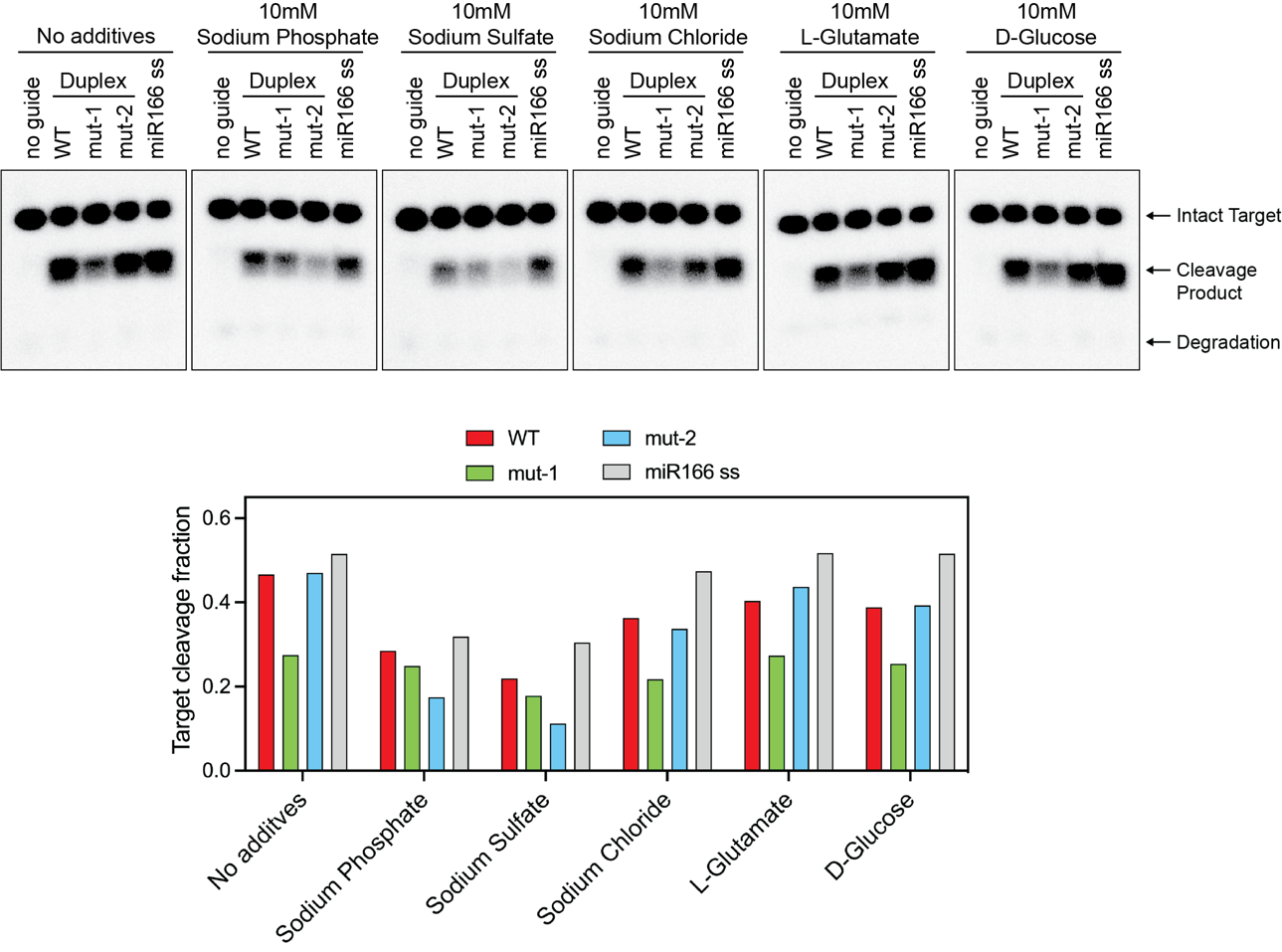


**Fig. S5. The effects of more compounds on AtAGO10-miRNA assembly reaction.** Dentaturing PAGE of target cleavage reactions using AtAGO10-miR166 complexes formed in the presence of various compounds (upper panel). Quantification of the cleavage reactions is shown in the lower panel.


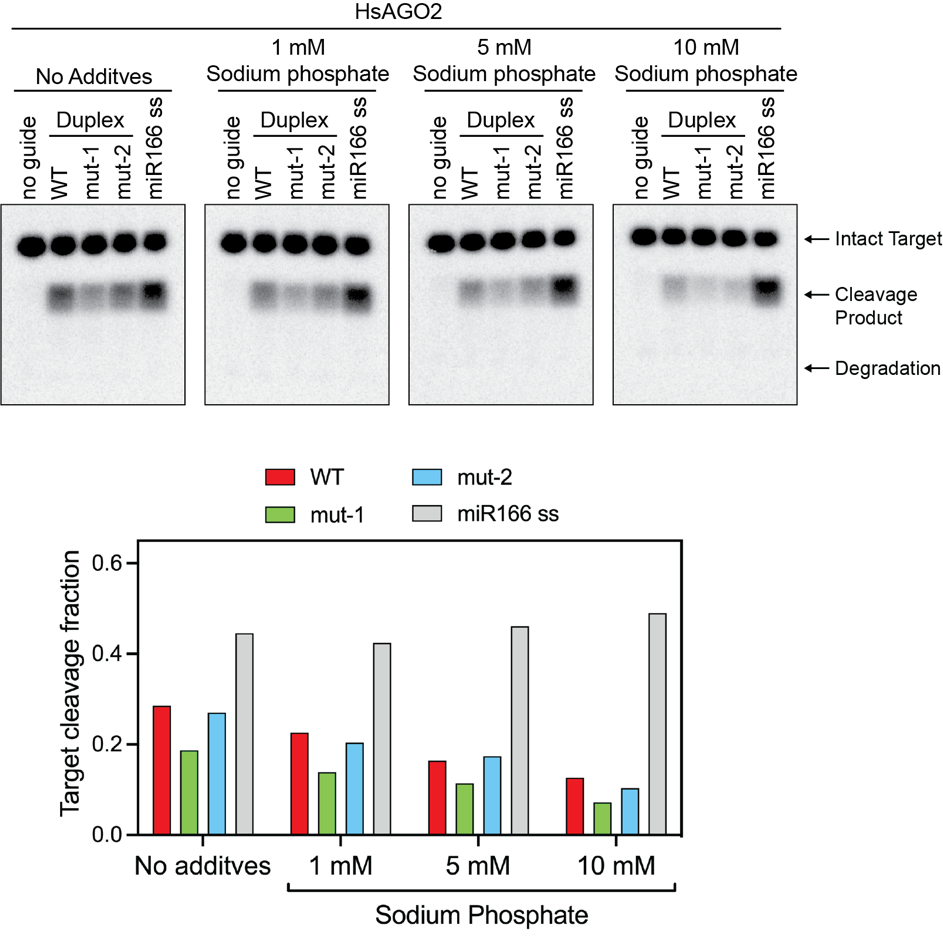


**Fig. S6. Phosphate inhibits HsAGO2-miRNA assembly but does not change duplex selectivity.** Denaturing PAGE of target cleavage reactions using HsAgo2-miR166 assembled in the presence of various concentrations of sodium phosphate (upper panel). The lower panel shows the quantification of bands in gels.

**
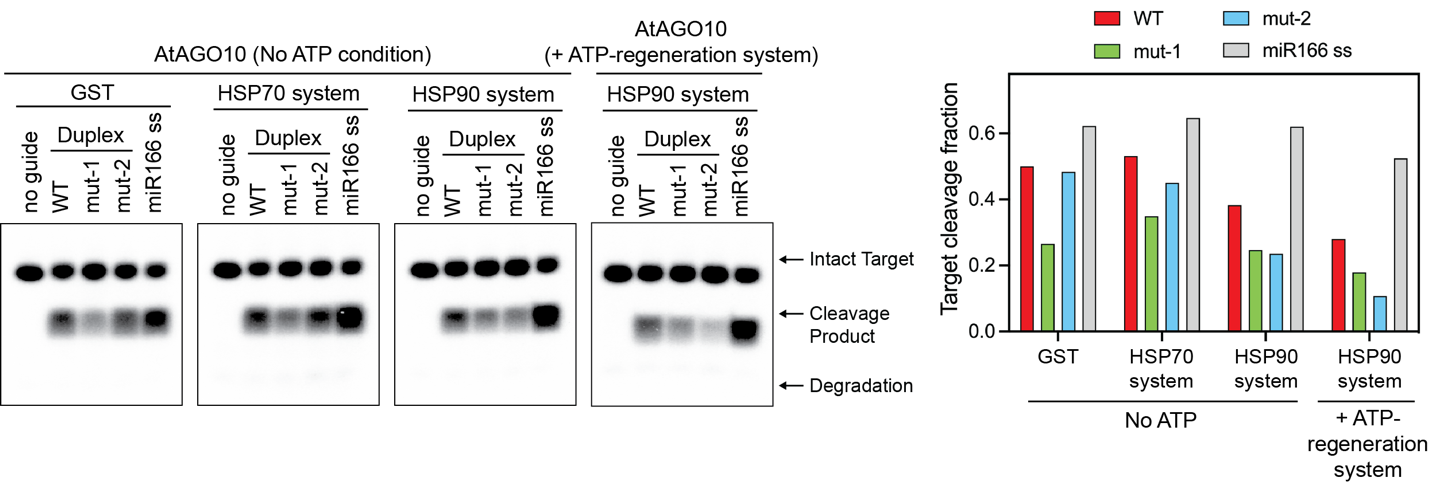
**

**Fig. S7. HSP90 system alone can inhibit the duplex loading into AtAGO10 without ATP in the system.** Denaturing PAGE of target cleavage reactions using AtAgo10-miR166 complexes assembled with or without chaperone systems and ATP regeneration (left panel). Quantification of bands on gels is shown in the right panel.


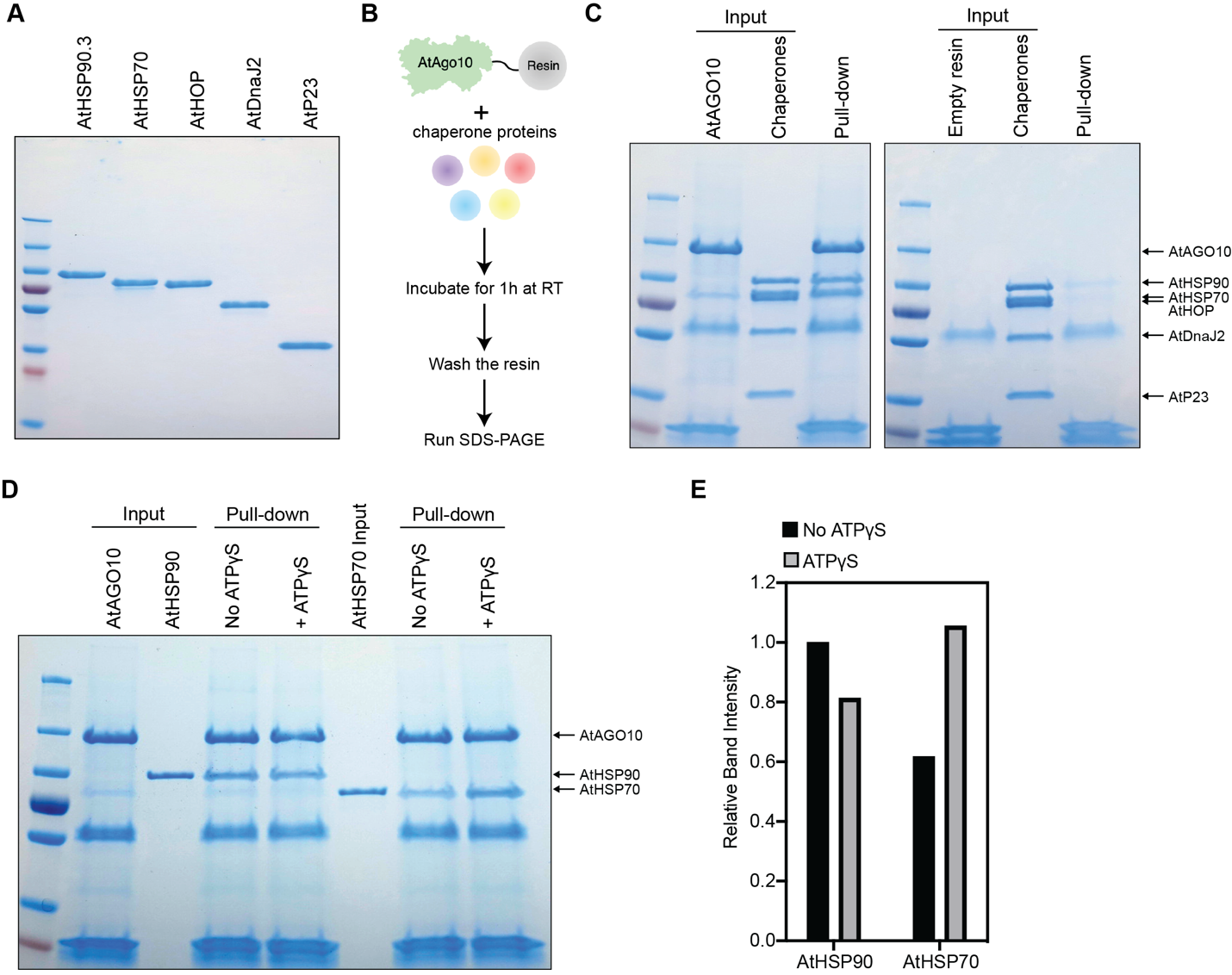


**Fig. S8. AtAGO10 directly interacts with AtHSP70 and AtHSP90. (A)** Coomassie-stained SDS PAGE of purified chaperone proteins used in this study. **(B)** Flow chart of the pull-down assay. **(C)** Coomassie-stained SDS PAGE of input and pulled down protein samples using immobilized AtAGO10. **(D)** Coomassie-stained SDS PAGE of pull-down of AtHSP90 and AtHSP70 by immobilized AtAgo10 ±ATPγS (non-hydrolyzable ATP analog). ATPγS enhanced pull-down of HSP70 and moderately reduced pull-down of HSP90. **(E)** Quantification of AtHSP90 and AtHsp70 bands in pull-down fraction in D. Intensities are normalized to the AtHSP90 band in no ATPγS pull-down sample.


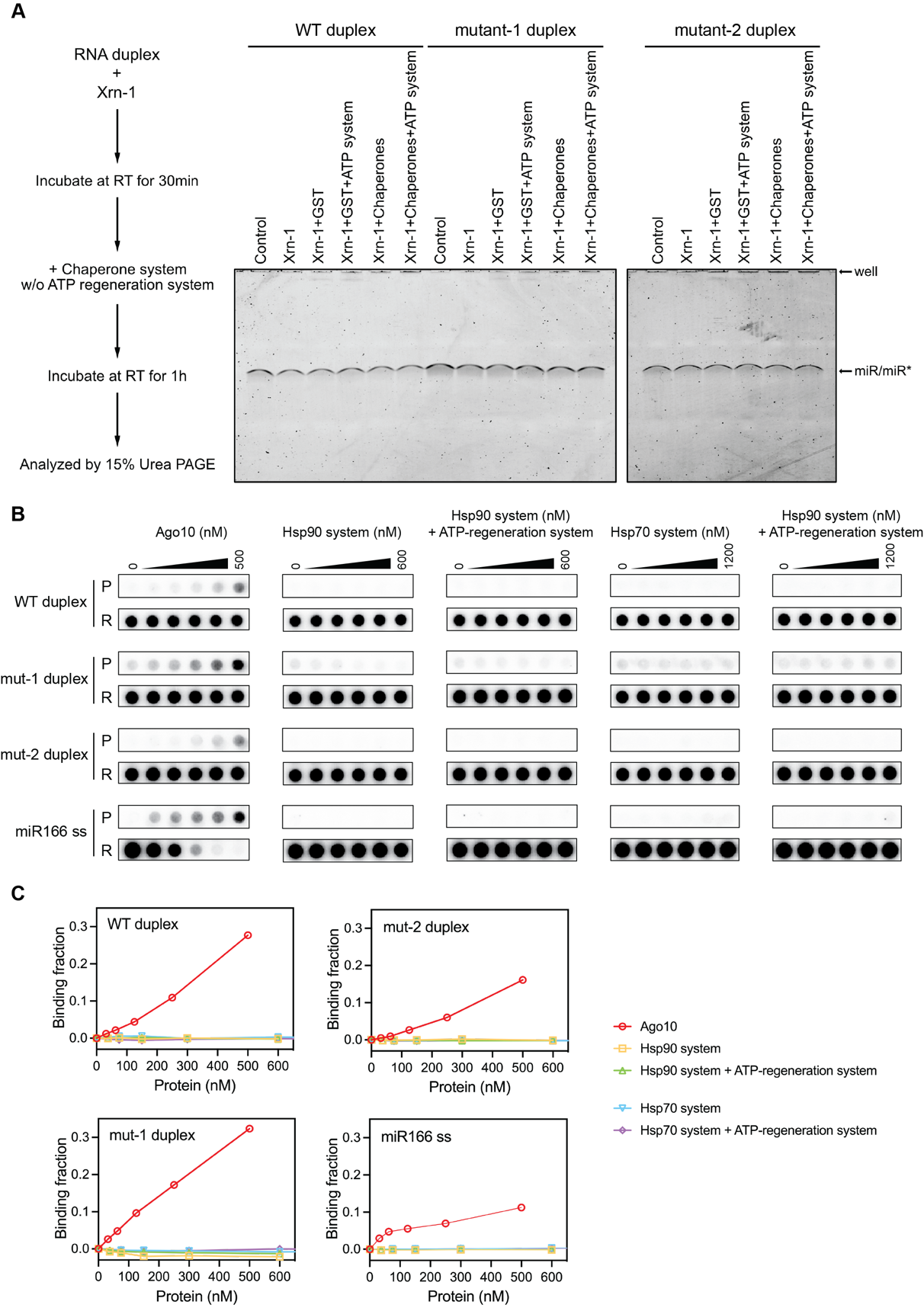


**Fig.S9. Chaperone system doesn’t affect guide RNA. (A)** The RNA stability test when incubated with chaperone proteins. **(B)** Dot-blot assay to detect the association between RNA and protein. **(C)** Quantification of RNA binding fraction in B.


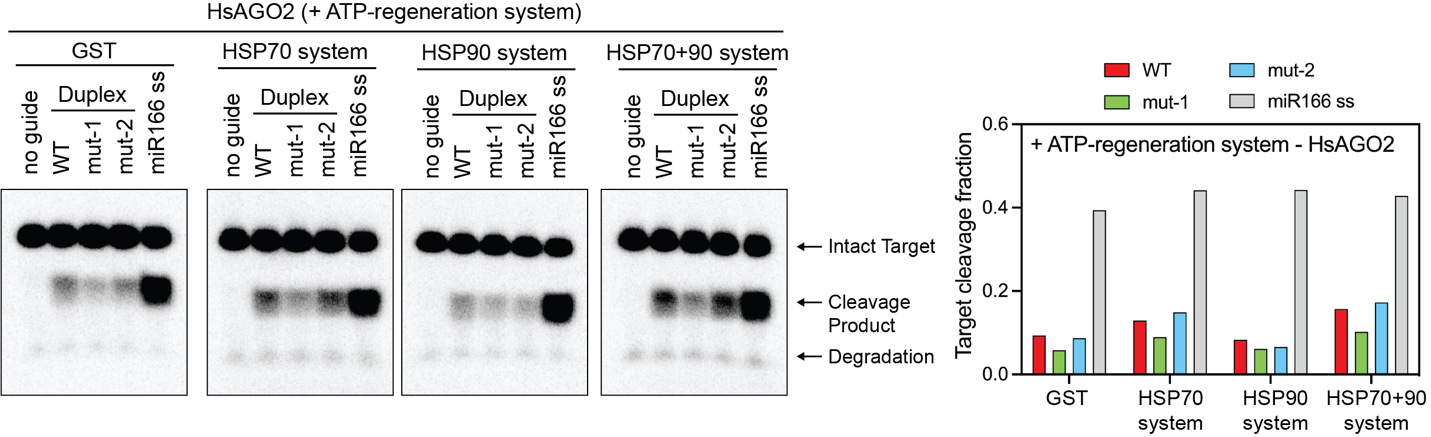


**Fig. S10. The effect of the chaperone systems on HsAGO2-miRNA assembly.** Data displayed as in Fig. 4.
